# Supplementary material for: The Perceptions of and Attitudes Toward Obesity in Bulgarian Adults with a BMI ≥ 25.0 kg/m2—An Exploratory Study
Source: Nutrients. 2025 Jan 21;17(3):373. doi: 10.3390/nu17030373 (PMC11820006; doi:10.3390/nu17030373)
Supplement: Supplementary file 1 [file nutrients-17-00373-s001.zip › nutrients-3388488-supplementary.pdf]

CARE FOR OVERWEIGHT/OBESITY
QUESTIONNAIRE

Screening

S1. Gender of the respondent?
Single answer

Male	1	
Female	2	

S2. What is your age?
Numeric

Below 25	1	END INTERVIEW
...	y/o	CONTINUE
Over 64	2	END INTERVIEW

S3. Do you or anyone else in your household work in any of the following areas?
Single answer

Marketing, incl. marketing research	1	END INTERVIEW
Advertising, PR, journalism	2	
Medical, pharmacological sectors	3	
None of the above	4	CONTINUE

S4. In which of the following segments does your household's monthly income fall?
Single answer

Up to BGN 1500	1	END INTERVIEW
BGN 1501 – 2400	2	
BGN 2401 – 3000	3	CONTINUE
BGN 3001 – 5000	4	
BGN 5001 – 10000	5	
Over BGN 10001	6	
DK /Refuse to answer	7	END INTERVIEW

S5. Are you concerned about your (body) weight or the weight of another member of your family?
Multiple

Yes, for mine	1	CONTINUE
Yes, for another adult member of my family	2	END INTERVIEW
Both for mine and other member of my family	3	CONTINUE
I don't feel concerned	4	End interview

Some of the questions in this survey are of a personal nature. They refer to health and overweight care.
Please note that the survey data will be processed anonymously.

S6. Do you want to continue with the interview?
Single answer

Yes, let's continue	1	CONTINUE
No, I would not participate in the survey	2	END INTERVIEW

For the purpose of statistics, we would like to ask you about your height and weight.

S7. Approximately how many kilograms are you currently?
Numeric

...	kg	Continue
DK /NA	2	END INTERVIEW

S8. Approximately how many centimeters is your height?
Numeric

...	CM	CONTINUE
DK /NA	2	END INTERVIEW

SectionA: Awareness of health condition

A1. Overall, how would you rate your health status?
Single answer
1 EXCELLENT
2 VERY GOOD
3 COMPARATIVELY GOOD
4 RATHER BAD
5 BAD

A2. Do you have chronic health disorders/ complaints - recurring over time, regardless of their degree of severity?
Single answer
1 Yes
2 No

A3. Which of the following chronic disorders/ complaints or diseases do you have?
Multiple, if A2=1
1 HIGH CHOLESTEROL
2 HIGH BLOOD PRESSURE
3 HIGH BLOOD SUGAR
4 HEART DISEASE
5 TYPE 2 DIABETES
6 JOINT PAIN OR OSTEOARTHRITIS
7 SLEEP APNEA / SNORING
8 KIDNEY DISEASE
9 LIVER DISEASE
10 TENSION, DEPRESSION OR OTHER DISORDER OF A PSYCHOLOGICAL NATURE
11 OTHER

A4. What do you think is the main reason for the appearance of these disease/ complaints? Are there other causes? Anything else?
Open-end, if A2=1
____

A5. What did you do the last time you felt unwell/ had a complaint, regardless of the type and character of thecomplaint?
Single answer
1 I CONSULTED A DOCTOR
2 I CONSULTED A PHARMACIST
3 I DID NOT CONSULT A SPECIALIST, BUT I WAS TAKING MEDICINE WITHOUT A PRESCRIPTION
4 I DID NOT CONSULT A SPECIALIST, BUT I TREATED MYSELF WITH TEA, HERBS, NUTRITIONAL SUPPLEMENTS
5 I TOOK NO ACTION

A6. I am going to read you some statements related to your personal views on health. Please, indicate to what extent do you agree or disagree with each of them using a 5-point scale where 1 is "completely disagree", 2 – "strongly disagree", 3 – "neither neither disagree nor agree", 4 – "strongly agree"and 5 - "completely agree".
Single answer per statement
1 I ONLY GO TO A DOCTOR DURING AN EXTREME CASE, WHEN THE PROBLEM BEGINS TO WORRY ME A LOT
2 I DON'T CARE ABOUT THE EXTRA KILOGRAMS AS LONG AS I FEEL GOOD
3 THE PROBLEM WITH THE EXTRA KILOGRAMS HAS A SIGNIFICANT IMPACT ON MY QUALITY OF LIFE
4 I TRY TO AVOID LARGE FINANCIAL COSTS FOR TREATMENT
5 WHEN POSSIBLE, I PREFER TO USE ALTERNATIVE TREATMENT METHODS (E.G. BASED ON HERBS AND OTHER PLANT EXTRACTS)
6 IN GENERAL, I TRUST DOCTORS AND FOLLOW THEIR RECOMMENDATIONS
7 MY OVERWEIGHT PROBLEM CAUSES DETERIORATION OF MY CHRONIC DISORDERS/DISEASES
8 I OFTEN FEEL GUILTY FOR NOT FOLLOWING A DIET AND EXERCISING
9 BEING OVERWEIGHT IS A DISEASE THAT DAMAGES THE WHOLE BODY, NOT A TEMPORARY CONDITION
10 I USUALLY HANDLE COLDS AND MINOR AILMENTS BY MYSELF, WITHOUT THE INTERVENTION OF A DOCTOR OR PHARMACIST

A7. How many extra kilograms do you think you have above those with which you would feel best?
Numeric
..... kg

A8. which of the following statements is closest to the attitude of your family and friends towards the extra kilograms you have?
Single answer
1 Sometimes they make disparaging words, mock me
2 They don't approve of my extra kilograms, although they don't talk about it
3 They accept me calmly, without disapproval and without interfering
4 They are ready to give me all the support i need to lose my weight
5 Other
6 DK

A9. What do you think are the main reasons for the extra kilograms you have? Please indicate the ones you consider as most important?
Multiple
1 Hormonal changes i slow metabolism
2 Treatment with hormone-based drugs
3 Presence of concomitant/associated diseases such as diabetes, etc.
4 Sedentary lifestyle
5 Lack of physical activity
6 Stress, depression
7 Genetic predisposition
8 Excessive food consumption, frequent eating
9 Excessive consumption of sweet foods and pastries
10 Poor quality of food products in the stores
11 Irregular eating
12 Other
13 DK / it's difficult to say

A10. Imagine that you decide to deal with the problem of extra kilograms permanently, to reduce them permanently. Who is responcible for solving the problem?
Single answer
1 I am fully responsible for fixing the problem permanently
2 I am primarily responsible and to a lesser degree, the specialist I am consulting
3 I and the specialist with whom I consult are equally responsible for dealing with the problem permanently
4 My specialist is primarily responsible for the permanent solution of the problem
5 My specialist is fully responsible for the permanent solution of the problem
6 DK/ I can't say

A11. Has a doctor ever drawn your attention to the extra kilograms you have on their own initiative?
Single answer
1 Yes
2 No

Section B: Living with Overweight and the patient's journey to tackling their problem

B1. Thinking of being overweight, to what extent do you agree or disagree with each of the following statements? Please, indicate to what extent do you agree or disagree with each of them using a 5-point scale where 1 is "completely disagree", 2 – "Strongly disagree", 3 – "Neither disagree nor agree", 4 – "strongly agree"and 5 - "completely agree".
Single answer per statement
1 The problem with being overweight is due to lifestyle, not genes
2 being Overweight is a health problem that needs to be treated
3 I am anxious about the topic of being overweight and avoid commenting on it
4 The main motive for losing the extra kilograms is the aesthetics of my external appearance
5 My extra kilos are affecting my self-esteem
6 I have used methods of reducing my body weight many times but without permanent success and I have given up on changing my weight
7 I would like to lose a fewof my extra kilos - as much needed soi can look and feel better

8 I definitely prefer methods of Losing body weight that are easy and do not require great efforts

B2. Which of the following methods and approaches of dealing with extra kilograms you have heard of, even if you haven't tried using them?*Multiple*
B3. And which methods and approaches of dealing with the extra kilograms you have ever tried?
Multiple, if checked on B2
B4. Which of these methods/approaches of dealing with extra kilograms you have used most often?
Multiple, if checked on B3
B5. Which methods and approaches of dealing with extra kilograms are you currently using?
Multiple, if checked on B2

LIST OF METHODS
1 Participation in a weight loss program of a fitness club
2 Weight loss products without a medical prescription, incl. medicines, food supplements, teas, etc.
3 Nutrition or diet (with/ without combination with physical activity)
4 Physical exercises and activity
5 Cosmetic procedures – skin tightening/lifting, slimming massage, etc.
6 Surgical procedures for weight loss such as liposuction
7 Painless injection therapy prescribed by a physician
8 Drugs prescribed by a doctor to reduce body weight/ excess kilograms
9 Prescribed medications for diseases other than being overweight that have an effect on weight loss
10 Other methods

B2 /11 I am unaware of any methods

B6. You mentioned that you haven't taken measures to deal with your extra weight ever. What are the main reasons for this decision?
Please indicate the most important reasons.
Multiple, if B2=11
1 I feel good enough with my weight
2 I have not encountered support from my closest relatives
3 I did not have enough information/ I did not know where to start
4 I did not have the financial resources
5 I don't think my weight affects my health
6 I don't trust the methods and ways I've heard of
7 I lack will /persistence for a good effect
8 Other

B7. How would you rate the efficiency of each weight management method you have tried? Please, indicate the level of efficiency of each method using a 5-point scale where 1 is "very inefficient method", 2 – "inefficient", 3 – "neither inefficient nor efficient", 4 – "efficient" and 5 - "very efficient method".
Single answer per row, if checked on B3
1 Participation in weight loss programs at a fitness clubs
2 Weight loss products without a medical prescription - supplements, teas, etc.
3 Nutrition or diet with/without combination with physical activity
4 Physical exercises and activity
5 Cosmetic procedures - , slimming massage, etc.
6 Surgical procedures for weight loss
7 Painless injection therapy prescribed by a doctor
8 Drugs prescribed by a doctor for weight loss
9 Medicines prescribed by a doctor for a disease other than being overweight that have an effect on reducing extra kilograms
10 Another method

B8. Have you ever consulted any of the following specialists to deal with your overweight on your own initiative?
Multiple
1 Doctor
2 Pharmacist
3 Nutritionist
4 Fitness instructor
5 Consultant in a beauty center
6 Other type of specialist/ please specify ....

7 I HAVE NOT CONSULTED WITH A SPECIALIST ABOUT MY BODY WEIGHT

B9. LAST TIME YOU MADE A DECISION AND TOOK MEASURES TO REDUCE YOUR WEIGHT, DID YOU CONSULT ANYONE AND IF YES, WHOM DID YOU ADDRESS?
MULTIPLE, IF B2≠11
1 I MADE THE DECISION AFTER PERSONAL CONSULTATION WITH A DOCTOR
2 I MADE THE DECISION AFTER CONSULTING A SPECIALIST IN THE FIELD, ALTHOUGH NOT A DOCTOR (NUTRITIONALIST, FITNESS INSTRUCTOR, BEAUTY CENTER CONSULTANT, ETC.)
3 I MADE THE DECISION AFTER GETTING INFORMATION FROM ELSEWHERE (FRIENDS, FAMILY, INTERNET, ETC.)
4 I DID NOT CONSULT ANYONE, I MADE THE DECISION MYSELF

B10. WHEN CONSULTING A DOCTOR - WHAT SPECIALTIST DID YOU CONSULT IN PERSON THE LAST TIME?
SINGLE ANSWER, IF B9=1
1 GENERAL PRACTITIONER (GP)
2 NUTRITIONIST
3 CARDIOLOGIST
4 GASTROENTEROLOGIST
5 ENDOCRINOLOGIST
6 INTERNAL DISEASES/ INTERNIST
7 OTHER TYPE OF SPECIALTY/ PLEASE, SPECIFY ....
8 I DON'T REMEMBER

B11. WITH WHAT KIND OF SPECIALIST OR NON-PHYSICIAN EXPERT DID YOU CONSULT IN PERSON THE LAST TIME?
SINGLE ANSWER, IF B9=2
1 PHARMACIST
2 NUTRITIONIST
3 FITNESS CONSULTANT/ INSTRUCTOR
4 CONSULTANT IN A BEAUTY CENTER
5 OTHER TYPE OF SPECIALIST/ PLEASE, SPECIFY ....
6 I DON'T REMEMBER

B12. THINKING OF MEDICATIONS FOR WEIGHT LOSS, WHAT IS THE FIRST THAT COMES TO YOUR MIND? WHAT ELSE CAN YOU THINK OF? ANYTHIING ELSE?
OPEN-END

____

B13. AND HAVE YOU EVER TAKEN A MEDICATION THAT HAD THE EFFECT OF LOSSING BODY WEIGHT?
MULTIPLE
1 YES, MEDICATION FOR WEIGHT REDUCTION
2 YES, MEDICATION WITH ANOTHER PURPOSE BUT WITH AN EFFECT ON WEIGHT REDUCTION
3 NO, I HAVE NEVER TAKEN A MEDICATION WITH AN EFFECT ON REDUCING BODY WEIGHT

B14. ARE YOU CURRENTLY TAKING SUCH MEDICATION?
SINGLE ANSWER
1 YES
2 NO

B15.WHAT WAS THE SPECIALTY OF THE DOCTOR WHO PRESCRIBED YOU THE LAST MEDICATION YOU HAVE TAKEN?
SINGLE ANSWER, IF B13=1 OR 2
1 GENERAL PRACTITIONER (GP)
2 NUTRITIONIST
3 CARDIOLOGIST
4 GASTROENTEROLOGIST
5 ENDOCRINOLOGIST
6 INTERNAL DISEASES/ INTERNIST
7 OTHER TYPE OF SPECIALTY/ PLEASE, SPECIFY ....
8 I DON'T REMEMBER

B16. HOW LONG WAS THE INTAKE DURATION OF THE LAST MEDICATION YOU HAVE TAKEN?
SINGLE ANSWER, IF B13=1 OR 2
1 LESS THAN A MONTH
2 2-3 MONTHS
3 4-6 MONTHS

4 7-12 MONTHS
5 1-2 YEARS
6 3-5 YEARS
7 6 AND MORE YEARS

Section C: Attitudes towards losing weight&using information channels

C1. DO YOU INTEND TO TAKE MEASURES TO REDUCE YOUR WEIGHT IN THE NEAR FUTURE?
SINGLE ANSWER
1 I HAVE ALREADY TAKEN SUCH MEASURMENTS
2 YES, I HAVE INTENTION
3 DK / I HAVEN'T DECIDED
4 I HAVE NO INTENTION

C2. HOW MANY KILOGRAMS FROM YOUR BODY WEIGHT YOU WOULD LIKE TO LOSE?
NUMERIC, IF C1≠4
… KG

C3. HOW MUCH TIME DO YOU THINK IT ISI REALISTIC TO LOSE THE EXCESS KILOGRAMS?
SINGLE ANSWER
1 1-3 MONTHS
2 4-6 MONTHS
3 7-9 MONTHS
4 10-12 MONTHS
5 13-18 MONTHS
6 19-24 MONTHS
7 25-36 MONTHS/2-3 YEARS
8 OVER 3 YEARS

C4. WHICH REASONS WOULD ENCOURAGE YOU MORE TO LOSE YOUR EXTRA KILOGRAMS - AESTHETIC OR HEALTH-RELATEDONES?
SINGLE ANSWER
1 AESTHETIC
2 HEALTH-RELATED

C5. HOW URGENT IS YOUR NEED TO LOSE WEIGHT TO IMPROVE YOUR HEALTH STATUS?
SINGLE ANSWER
1 VERY URGENT
2 RATHER URGENT
3 NEITHER URGENT NOR NON-URGENT
4 RATHER NOT URGENT
5 NOT AT ALL URGENT

C6. NO MATTER HOW URGENT, WHICH OF THE FOLLOWING YOU ARE MOST LIKELY TO CARRY OUT AS TO REDUCE YOUR WEIGHT?
SINGLE ANSWER
1 I WILL DO IT MYSELF WITH THE HELP OF THE INTERNET AND DIGITAL APPS
2 I WILL CONSULT BOTH THE INTERNET AND A SPECIALIST
3 I WILL CONTACT A SPECIALIST WHOSE ADVICE I WOULD FOLLOW
4 DK / I CAN'T SAY

C7. HOW OFTEN DO YOU USE EACH OF THE FOLLOWING MEDIA?
SINGLE ANSWER PER CHANNEL
1 EVERY DAY | 2 2-3 TIMES A WEEK | 3 ONCE A WEEK | 4 LESS OFTEN| 5 DO NOT USE
1 RADIO
2 TELEVISION
3 INTERNET
4 NEWSPAPERS AND MAGAZINES
5 SPECIALIZED ONLINE EDITIONS FOR HEALTH AND BEAUTY
6 SPECIALIZED NEWSPAPERS AND MAGAZINES FOR HEALTH AND BEAUTY

C8. What information sources do you use to learn about current weight management methods and approaches?
Multiple
C9. And which of these sources of information do you mostly trust?
Single answer

LIST OF INFORMATION SOURCES
1 Relatives and friends
2 A socially influential person who shares his experience
3 Doctor
4 Pharmacist
5 Nutritionist (not necessarily a doctor)
6 Fitness specialist/ instructor
7 Specialist/ consultant in a beauty center
8 Mass electronic media – television and radio
9 Articles in the mass press and magazines
10 Articles in specialized health journals
11 Social media on the internet
12 Corporate website - pharma manufacturer, clinic, fitness
13 Other source of information
14 None/ I do not use sources on the topic

C10. How often do you get information about weight loss from the mass media - television, radio, press?
Single answer, if C9≠14
1 Once a week and more often
2 2-3 times a month
3 Once a month
4 Once every few months
5 Once a year
6 Very rarely at sporatic times
7 I do not get information on the topic from the mass media

C11. How often do you get information about weight loss on the internet?
Single answer, if C9≠14
1 Once a week and more often
2 2-3 times a month
3 Once a month
4 Once every few months
5 Once a year
6 very rarely at sporatic times
7 I do not get information on the topic from internet

C12. What is the specialty of the medical doctor you would trust the most when wishing to be prescribed a medication for weight loss?
Single answer
1 General practitioner (GP)
2 Nutritionist
3 Cardiologist
4 Gastroenterologist
5 Endocrinologist
6 Internal diseases/ internist
7 Other type of specialty/ please, specify ....
8 I don't remember

C13. How likely are you to use a prescribed weight loss medication?
Single answer
1 I will definitely not use such medicine
2 I will most likely not use such medicine
3 I may use it but i may not
4 I will most likely use it
5 I will definitely use it

Section D: Additional demographic data

D1. Education level of participant?
Single answer
1 University
2 Secondary
3 Primary and lower

D2. Who co-habitates with you in your household currently?
Multiple
1 Alone/No one
2 Partner
3 My kids
4 My parents
5 Other family members
6 Other

D3. How many children under the age of 18 live in your household?
Numeric
____
99 No children under 18 live in our household

D4. Occupation of participant?
Single answer
1 Entrepreneur/ self-employed
2 Worker/ employee
3 Student
4 Housewives/ maternity leave
5 Unemployed/ temporary unemployed

D5. What Urban Settlement Type do participants live in? Town/Village etc.

D6. Living in which District?

THAT WAS THE LAST QUESTION!
THANK YOU FOR YOUR TIME!
